# A Rapid Terrestrial Laser Scanning Method for Coastal Erosion Studies: A Case Study at Freeport, Texas, USA

**DOI:** 10.3390/s19153252

**Published:** 2019-07-24

**Authors:** Lin Xiong, Guoquan Wang, Yan Bao, Xin Zhou, Kuan Wang, Hanlin Liu, Xiaohan Sun, Ruibin Zhao

**Affiliations:** 1 Department of Earth and Atmospheric Sciences, University of Houston, Houston, TX 77204, USA; 2 The Key Laboratory of Urban Security and Disaster Engineering of Ministry of Education, Beijing University of Technology, Beijing 100124, China; 3 Institute of Urban Smart Transportation and Safety Maintenance, College of Civil Engineering, Shenzhen University, Shenzhen 518060, China; 4 School of Civil Engineering, Tianjin Chengjian University, Tianjin 300384, China; 5 School of Geology and Geomatics, Tianjin Chengjian University, Tianjin 300384, China

**Keywords:** coastal erosion, DEM, georeferencing, GPS, OPUS, LiDAR, registration, TLS

## Abstract

Terrestrial laser scanning (TLS) has become a powerful data acquisition technique for high-resolution high-accuracy topographic and morphological studies. Conventional static TLS surveys require setting up numerous reflectors (tie points) in the field for point clouds registration and georeferencing. To reduce surveying time and simplify field operational tasks, we have developed a rapid TLS surveying method that requires only one reflector in the field. The method allows direct georeferencing of point clouds from individual scans to an East–North–Height (ENH) coordinate system tied to a stable geodetic reference frame. TLS datasets collected at a segment of the beach–dune–wetland area in Freeport, Texas, USA are used to evaluate the performance of the rapid surveying method by comparing with kinematic GPS measurements. The rapid surveying method uses two GPS units mounted on the scanner and a reflector for calculating the northing angle of the scanner’s own coordinate system (SOCS). The Online Positioning User Service (OPUS) is recommended for GPS data processing. According to this study, OPUS Rapid-Static (OPUS-RS) solutions retain 1–2 cm root mean square (RMS) accuracy in the horizontal directions and 2–3 cm accuracy in the vertical direction for static observational sessions of approximately 30 min in the coastal region of Texas, USA. The rapid TLS surveys can achieve an elevation accuracy (RMS) of approximately 3–5 cm for georeferenced points and 2–3 cm for digital elevation models (DEMs). The elevation errors superimposed into the TLS surveying points roughly fit a normal distribution. The proposed TLS surveying method is particularly useful for morphological mapping over time in coastal regions, where strong wind and soft sand prohibit reflectors from remaining strictly stable for a long period. The theories and results presented in this paper are beneficial to researchers who frequently utilize TLS datasets in their research, but do not have opportunities to be involved in field data acquisition.

## 1. Introduction

**Figure 1:**
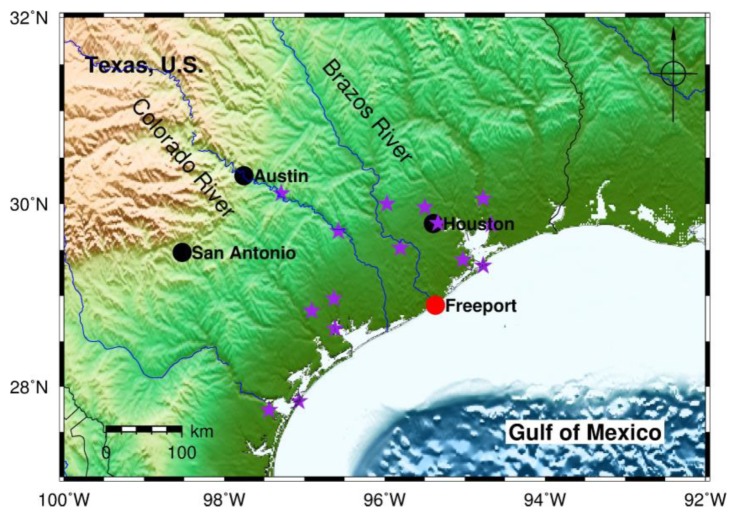
Map showing the location of the study area in Freeport, Texas, USA. Purple stars represent Continuously Operating Reference Stations (CORS) used by the Online Positioning User Service (OPUS) for solving the positions of the scanner according to 15 to 30-min static GPS observations.

Terrestrial laser scanning (TLS), also known as ground-based light detection and ranging (LiDAR), has become a powerful research tool for tracking ground surface deformation over time and space, such as landslide movement [1,2,3,4,5], glacial movement [6,7,8,9], faulting [10,11], and earth fissures [12]. Dense point clouds from TLS surveys provide fundamental datasets for deriving high-resolution and high-accuracy bare-earth digital elevation models (DEMs), which are essential for topographic and geomorphic studies. Recently, TLS has been frequently applied for studying coastal erosion problems through repeated surveys [13,14,15]. Coastal erosion has exerted a continuing threat to the Texas Gulf coastal area during the past three decades [16]. This study investigated the erosion problem in the Freeport beach and dune area, where the Brazos River enters the Gulf of Mexico (Figure 1).

Laser measurements require line-of-sight (LOS). An object that is not in the sight of a TLS scanner will not be scanned. Since natural topography is usually not flat and smooth, numerous scans are required from different directions to fully scan field objects. For example, for scanning a landslide in Puerto Rico with an approximate area of 150 m by 200 m, 25 individual scans were conducted to fully cover the landslide surface [2]. Conventional TLS survey methods apply two steps to align point clouds from individual scans to a common geodetic coordinate system. The original coordinates of laser points from an individual scan are referred to as the scanner’s own coordinate system (SOCS). The first step is to link the point clouds from individual scans to a project coordinate system (PROCS) in the order of field scanning. This process is called registration. In practice, the SOCS of the first scan is often set as the PROCS. Registration is performed in the field by tie points (reflectors). In order to track minor topographic changes over time, periodically-repeated TLS surveys are performed over years (e.g., one decade). These point clouds are referred to different PROCS. So, the second step is to transform PROCS to a common geodetic reference frame according to GPS-measured positions at a few reflectors, such as the North American 1983 Datum (NAD83), for the case study. This process is called georeferencing. 

The theories behind the registration and georeferencing are the same: using a seven-parameter Helmert transformation algorithm to align two three-dimensional (3D) Cartesian coordinate systems. In general, registration focuses on stitching point clouds from individual scans together according to certain common points or topographic features, while georeferencing focuses on aligning point clouds to a regional geodetic coordinate system according to GPS measurements. A minimum of three common points (reflectors) is required to align two 3D Cartesian coordinate systems, but a greater number of common points will increase the numerical stability and reliability of the transformation. 

**Figure 2:**
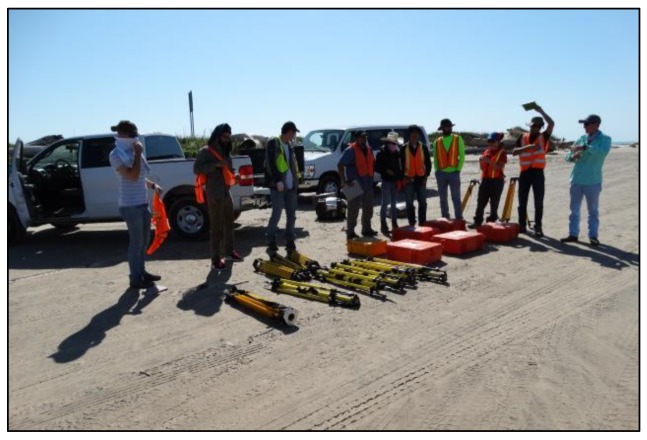
Heavy equipment sets used for mapping the Freeport beach and dune area with the conventional terrestrial laser scanning (TLS) surveying method.

In practice, five or more common reflectors are often used for deriving key parameters for each coordinate transformation, and ten or more reflectors are used in a field project to make sure two neighbor scans share at least three common reflectors. As a consequence, conventional TLS surveys require numerous tripods, reflectors, GPS units, and significant manpower in the field for point clouds registration and georeferencing. For our previous TLS survey projects at the Freeport beach area [17] and the Puerto Rico landslide site [2,18], approximately ten reflectors and five GPS units were used in the field. High-resolution and high-accuracy TLS surveys can be time consuming. Figure 2 exhibits tripods and reflectors (in red boxes) that we used in earlier years (before 2015) for scanning the beach-dune area in Freeport, Texas. It took quite a lot of time to transport, set up, identify, rescan (fine-scan), and relocate reflectors in the field. Furthermore, the conventional surveying method requires reflectors to remain stable for several hours while being scanned from different scan sites. Unfortunately, the soft sand and strong wind in coastal environments make it a challenge to keep reflectors stationary for several hours. This study aims to develop a rapid and high-accuracy TLS surveying method that only requires one reflector and two GPS units in the field. These two GPS units are used to survey the positions of the scanner and reflector, and in turn, to estimate the northing angle of the SOCS. The Online Positioning User Service (OPUS) operated by the National Geodetic Survey (NGS) is recommended for GPS data processing. The performance of OPUS Rapid-Static (OPUS-RS) in the coastal region of Texas, USA is investigated in this study.

## 2. Materials and Methods

### 2.1. Study Area

**Figure 3:**
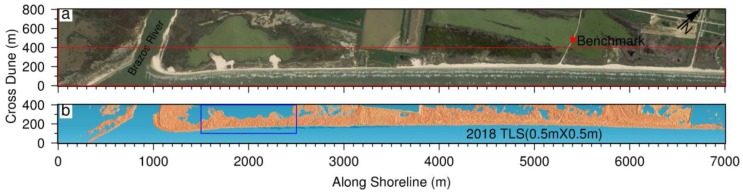
(**a**) A Google Earth image showing the beach–dune–wetland area in Freeport, Texas; (**b**) hill-shaded bare earth topographic map derived from TLS datasets collected in May 2018. The coordinate system for the image and point clouds was rotated 40 degrees counter-clockwise for better demonstration. The red star is the location of the U.S. Geological Survey (USGS) permanent benchmark. The blue rectangle indicates the area for evaluating the performance of the rapid surveying method.

**Figure 4:**
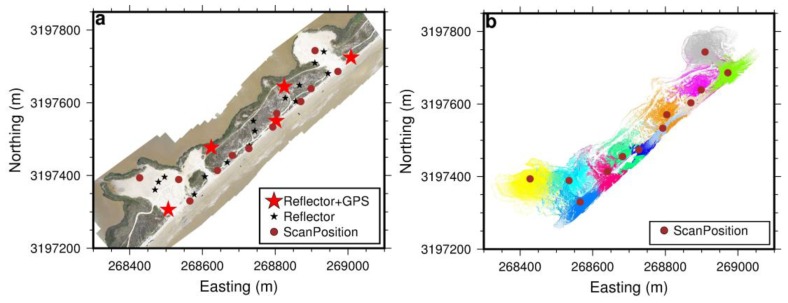
(**a**) Locations of reflectors and scanners used for collecting TLS datasets for the case study on 29 April 2018; (**b**) point clouds from 12 individual scans. The coordinates refer to the Universal Transverse Mercator (UTM) 15R system.

The authors have been monitoring coastal erosion problems in Freeport, Texas, since the summer of 2013. A 7-km long and 0.5-km wide beach–dune–wetland segment has been periodically scanned twice per year, using the rapid surveying method since 2015 and the conventional surveying method in earlier years (Figure 3). A Riegl VZ-2000 scanner is used in the field. In order to evaluate the performance of the rapid surveying method, we collected both TLS and GPS datasets within an approximately 800 m long and 300 m wide segment of the beach and dune area, as marked in Figure 3b, on 29 April 2018. The TLS datasets were registered and georeferenced using both the conventional and rapid surveying methods. In total, 12 scans were conducted to map the test area (Figure 4a). Twenty reflectors were scanned during the field surveys, including 15 standard cylinder reflectors (10 cm in diameter and 10 cm in height) manufactured by Riegl and five larger reflectors (20 cm in diameter and 25 cm in height) designed by the Geodesy Lab at the University of Houston. Figure 4b depicts data coverage of each scan. Although the scanner has the capability to receive laser returns as far as 2 km away, the majority of returns are within a range of 300 m from the scanner in the flat beach area. Accordingly, the accuracy assessment performed in this study is limited to point clouds within 300 m to the scanner in this study. In general, the accuracy of TLS measurements beyond 300 m of the scanner could be slightly worse.

### 2.2. Accuracy of TLS Range Measurements 

**Figure 5:**
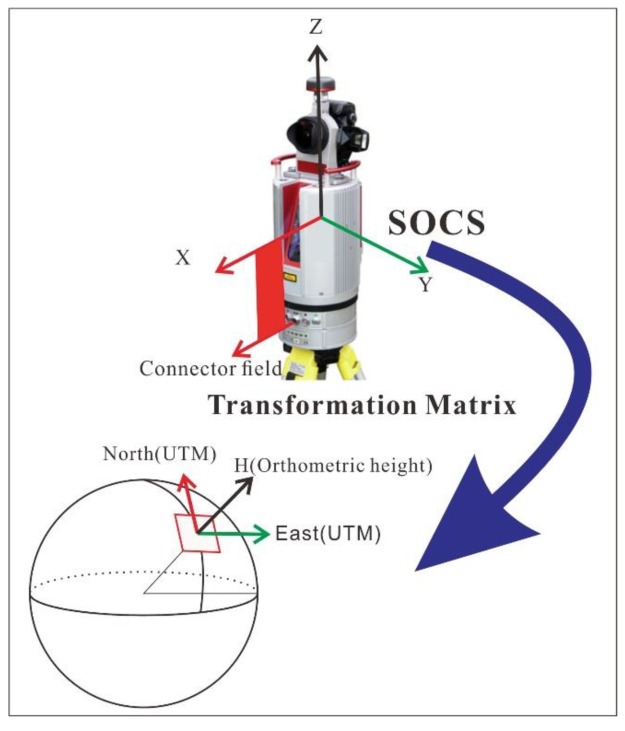
A conceptual sketch illustrating the georeferencing process employed in the rapid surveying method. The rapid surveying method directly transforms TLS points from the scanner’s own coordinate system (SOCS) to the regional geodetic coordinate system (ENH: East–North–Height).

TLS points are initially positioned with respect to a scanner-intrinsic spherical coordinate system consisting of one horizontal rotation angle and one vertical altitude angle of the laser source at the moment the laser pulse is sent out. The spherical coordinates are transformed into a 3D right-handed Cartesian coordinate system (XYZ) with respect to the SOCS (Figure 5). For the Riegl VZ-2000 scanner, the x-axis of the SOCS is aligned with the direction of scanner’s cable connector port, the y-axis is perpendicular to the x-axis in the plane which is parallel to the scanner’s bottom plate, and the z-axis is perpendicular to the x–y plane. According to numerous technical investigations on laser scanners, the error of intrinsic coordinates with respect to the spherical coordinate system of the scanner is dominated by uncertainties of the laser rangefinder in scanners [19,20]. Our previous investigations indicate the precision of the laser rangefinder is at a level of 1 cm within a range of 300 m in the field [17]. This conclusion was further confirmed by the datasets obtained for the case study. 

In this study, the RiSCAN PRO 2.0 (http://www.riegl.com/products/software-packages/riscan-pro), the companion software package for Riegl’s 3D laser-scanners, was utilized to manage field data acquisition, registration, georeferencing, and the basic process. The primary process included removing outliers, octree filtering, and terrain filtering. The size of the cabin of the octree filtering was 3 cm in the horizontal directions and 1 cm in the vertical direction. The pre-processed coordinates were exported into the American Standard Code for Information Interchange (ASCII) format. The ASCII files were further processed with the Generic Mapping Tools (GMT) software package [17,21]. GMT is a widely used open source software in the geoscience community for processing and plotting geographic data and generating DEMs [22].

Table 1 illustrates the comparison of scanner-to-reflector distances measured by GPS and TLS. The TLS-derived distance was provided by the RiSCAN PRO software. The GPS-derived distance was calculated from observations of two GPS antennas, mounted on the scanner and a reflector. The common observation period of two GPS units is approximately 25 min. A carrier-phase double-difference (DD) method employed in a commercial software package Topcon Tools (Version 8.1) was used to calculate the GPS-derived distance. According to our previous investigations, the point-to-point distance derived from 25-min GPS observations would retain the accuracy of a few millimeters for baselines less than one kilometer [23,24]. Table 1 indicates that the agreement between the GPS-derived scanner-to-reflector distance and TLS-derived scanner-to-reflector distances was at a level of a few millimeters within a distance of 300 m. That is to say, the range-accuracy of the laser rangefinder in the scanner was at a sub-centimeter level. The sub-centimeter errors were much smaller than the total error budget of the georeferenced TLS points, which was at a level of approximately 3–5 cm, according to this study. Accordingly, this investigation omitted the intrinsic errors of the scanner and focused on errors related to registration and georeferencing.

### 2.3. Georeferencing Method

The essence of georeferencing is to transform point clouds from XYZ-coordinates with respect to a SOCS or a PROCS to a regional geodetic coordinate system. In surveying and geodesy communities, a geodetic coordinate system is often described by easting (E), northing (N), and height (H). In practice, E and N coordinates are often aligned to the easting and northing coordinates of the Universal Transverse Mercator (UTM). The study area was located in UTM Zone 15R. *H* is often aligned to an orthometric height system, the North American Vertical Datum of 1988 (NAVD88) for the case study. The value of the orthometric height was calculated according to the GEOID12B model [25]. In North America, the UTM coordinates are derived from the geocentric 3D Cartesian coordinates of the NAD83 (2011) geodetic reference frame [26]. A stable site in the Texas coastal region retains a steady horizontal velocity of approximately 2 mm/year and a vertical velocity below 1 mm/year with respect to NAD83 (2011) [27,28,29]. The accumulate displacements, or the instability of the regional reference frame, would be at a couple of centimeters level over a decade, which is below the expected accuracy for most TLS surveying projects. Thus, the ENH (East–North–Height) coordinate system aligned to UTM and NAVD88 provided a stable reference for precisely tracking topographic changes in the Texas coastal region within a time span of one decade.

There are several classical methods for coordinate transformation between two Cartesian coordinate systems. The Helmert transformation is the one that is frequently used in geodesy to produce distortion-free transformations between two 3D Cartesian coordinate systems. The transformation requires seven parameters: three translations ($T_{x}$, $T_{y}$, and $T_{z}$), three rotations ($R_{x}$, $R_{y}$, and $R_{z}$), and a scale factor (µ). The coordinate transformation from the SOCS’s XYZ-coordinates to ENH-coordinates can be performed by the following equation:

(1) $$\left[\begin{array}{c} E\\ N\\ H \end{array}\right]=\left[\begin{array}{c} T_{x}\\ T_{y}\\ T_{z} \end{array}\right]+\mu \times \left[\begin{array}{ccc} 1 & -R_{z} & R_{y}\\ R_{z} & 1 & -R_{x}\\ -R_{y} & R_{x} & 1 \end{array}\right]\times [\begin{array}{c} X\\ Y\\ Z \end{array}].$$

The UTM system is a map projection of the curved Earth surface. Coordinate projections often cause distant distortions. For example, for two scan positions SP03 and SP07 (Table 1), their 3D distance is 305.370 m with respect to the geocentric coordinate system (XYZ) associated with NAD83 (2011) and 305.450 m with respect to the ENH coordinate system. The difference between the two distance measurements is 8.0 cm, which can be even larger if the distance is longer. The distortion coefficient of the 3D distance measurements from XYZ to ENH in the study area is approximately 1.00026. Accordingly, the scale factor µ was set as 1.00026 in the study area. $T_{x}$, $T_{y}$, and $T_{z}$ can be derived from the GPS measurements mounted on the scanner. $R_{x}$, $R_{y}$, and $R_{z}$ are three rotations of the three axes of the SOCS with respect to three axes of the ENH coordinate system. For a static TLS survey, $R_{x}$ and $R_{y}$ are two tilt angles of the scanner relative to the vertical plane; $R_{z}\;$is the northing angle of the scanner’s x-axis. 

One single-frequency GPS unit, two built-in inclination sensors, and one electronic compass have become a standard configuration of modern terrestrial laser scanners, such as Riegl-VZ scanners and Leica ScanStation P50/P40/P30 scanners. The GPS unit is able to provide the location information of the scanner, specifically, the location of the origin of the SOCS with respect to a global reference frame. The onboard inclination sensors measure two tilt angles ($R_{x}$, $R_{y}$) of the scanner. The onboard compass estimates the northing angle of the scanner’s x-axis ($R_{z}$). Thus, point clouds from individual scans can be directly georeferenced to the ENH coordinate system, according to Equation 1. The approach for georeferencing using measurements from onboard sensors is called direct-georeferencing, which has been discussed by several publications, and the overall positional accuracy varies from meters to decimeters [30,31,32]. These previous investigations mostly targeted decimeter-level surveying accuracy, and the direct-georeferencing mainly was utilized as an initial alignment of individual scans. Other data-driven algorithms were utilized to refine the pre-georeferenced point clouds to achieve higher positional accuracy, such as the Iterative Closest Point (ICP) method [33,34,35]. Direct-georeferencing methods have been rarely applied for TLS surveys targeting morphological studies that require positional accuracy at a few centimeters. 

**Figure 6:**
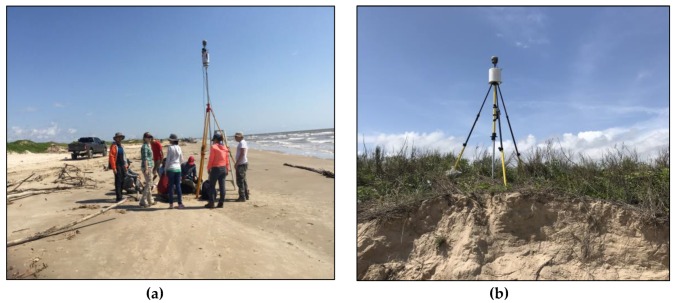
Photos showing field equipment used by the rapid TLS surveying method in the field. (**a**) A Riegl VZ-2000 scanner with a co-mounted Trimble R10 GPS unit; (**b**) a cylinder reflector with a co-mounted Trimble R10 GPS unit.

A common concern about direct-georeferencing is the accuracy and precision of these onboard sensors. In general, the positional accuracy of a single-frequency GPS unit is at a meter-level. Thus, the onboard GPS is not able to provide the positional accuracy that is needed for centimeter-accuracy TLS surveys. We propose to use a dual-frequency GPS unit (Trimble R10) to replace the onboard single-frequency GPS unit. For the Riegl VZ-2000 scanner (Figure 6a), the precision of the onboard inclinations is 0.008 degrees, according to the manufacturer, which could result in a vertical error of approximately 4 cm in georeferenced point clouds at a range of 300 m. The precision of the onboard compass is one degree. A one-degree uncertainty on the rotation of the z-axis of the scanner could result in a horizontal positional error up to 5.24 m at a range of 300 m. The horizontal errors at a few meters are obviously too large for centimeter-accuracy TLS surveys. Furthermore, the field performance of the onboard electronic compass can be biased by metallic materials on the tripod. As a consequence, the “northing angle” ($R_{z}$) measurement from the onboard compass cannot be directly used for high-accuracy TLS surveys. We developed a backsighting method that utilized two dual-frequency GPS units mounted on the scanner and a reflector to estimate the northing angle ($R_{z}$) (Figure 6a,b). The backsighting method only used the *E* and *N* coordinates derived from GPS measurements at the reflector and the scanner to calculate $R_{z}$. The field performance of the TLS and GPS integrated surveying method will be evaluated in the following sections.

### 2.4. GPS Data Processing Method

As mentioned earlier, the rapid surveying method utilized seven parameters for georeferencing. Four of these seven parameters were derived from static GPS observations on the scanner and the reflector—$T_{x}$, $T_{y}$, $T_{z}$, and $R_{z}.$ Thus, the accuracy of GPS measurements is fundamental for achieving high-accuracy TLS surveys. GPS data processing can be troublesome for TLS mapping projects, particularly for repeated TLS surveys across a long time span (e.g., over a decade). The reference frame transformation from a global geodetic reference frame to a regional reference frame could be complex and confusing even for professional surveyors. The alignment from an ellipsoid height system to an orthometric height system and projection from an earth-centric Cartesian coordinate system to a plane geographic coordinate system (e.g., UTM) can also be confusing for non-expert geodesy users. Fortunately for GPS surveying in the USA, the NGS provides OPUS for GPS data positioning and coordinate transformations. OPUS is a free, automated, and Web-based GPS post-processing utility that delivers accurate and reliable positional coordinates. Users do not need to set up reference stations in their working area and do not need to install any GPS-processing software packages on their computers. Practicing surveyors, engineers, and researchers with basic GPS knowledge are able to process GPS data using OPUS with little training. Thus, OPUS has become one of the most useful tools that NGS provides to the surveying, engineering, and academic communities [36]. 

OPUS offers two approaches, OPUS Static (OPUS-S) and OPUS Rapid-Static (OPUS-RS), for solving a static position. OPUS-S is used for observations longer than two hours; OPUS-RS is used for observations longer than 5 min but less than 2 h. OPUS-S solves a position using three single baselines from three known Continuously Operating Reference Stations (CORS). OPUS-RS solves positions for shorter sessions (<2 h) by interpolating the troposphere and ionosphere over the user’s point, using up to nine nearby CORS. Detailed information about CORS and OPUS may be consulted in a number of articles included in a monograph edited by Soler [37], and recent articles published by Soler and Wang [26] and Gillins, et al. [38].

According to our field experience, it takes approximately 5 min to set up equipment at a scan site, 15 min for the scanner to finish a panorama scan, and 10 min for identifying, rescanning the reflector, and packing up. The GPS unit mounted on the scanner is able to collect about 25 min of static data at a scan site. In practice, the reflector is often set up at a relatively high site and can be used for four or more scans. Thus, the GPS unit on the reflector often collects over two hours of data. GPS data collected on the scanner and reflector can be processed by OPUS-RS and OPUS-S, respectively. The performance of OPUS-S within the greater Houston region has been investigated by Wang and Soler [36] and Wang, et al. [39]. OPUS-S solutions are able to retain below 1.5 cm vertical accuracy and better horizontal accuracy for two-hour or longer data sessions in the greater Houston region. The accuracy could be further improved with longer observational periods [40]. However, the performance of OPUS-RS in the Texas coastal region has not been fully investigated. The accuracy of OPUS-RS solutions in coastal areas could be degraded by the poor geometry of available CORS stations [41]. Since the accuracy of OPUS-RS solutions ultimately affects the positional accuracy of georeferenced TLS points, the positional accuracy of OPUS-RS solutions in the Texas coastal region was assessed in this study. A brief report of the results is presented in the following section. 

## 3. Results

### 3.1. Accuracy of OPUS-RS 

**Figure 7:**
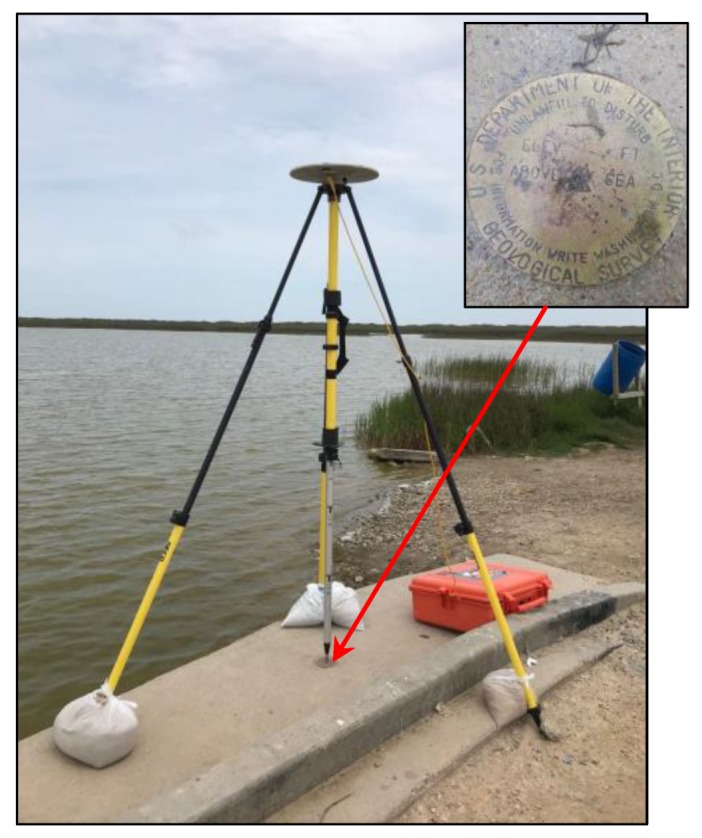
A photo showing the U.S. Geological Survey (USGS) permanent benchmark and the setting up of a reference station for kinematic GPS mapping in the beach area.

The U.S. Geological Survey (USGS) has been conducting repeated coastal surveys in Texas coasts for several decades. A permanent surveying benchmark was installed on a concrete bridge near the Freeport beach (Figure 7). The benchmark is approximately two kilometers away from the study site (Figure 3a). Thirty GPS surveys have been conducted at the benchmark site during the past five years. Each campaign lasted approximately 4 to 6 h. In order to evaluate the accuracy (repeatability) of OPUS-RS solutions, these datasets were split into 15-min segments and 30-min segments. In total, 618 files with 15-min data and 276 files with 30-min data were submitted to OPUS-RS. The locations of those reference stations used by OPUS-RS are marked in Figure 1. OPUS-RS is occasionally not able to solve a solution, mostly because of the deficiency of reference data. 

**Figure 8:**
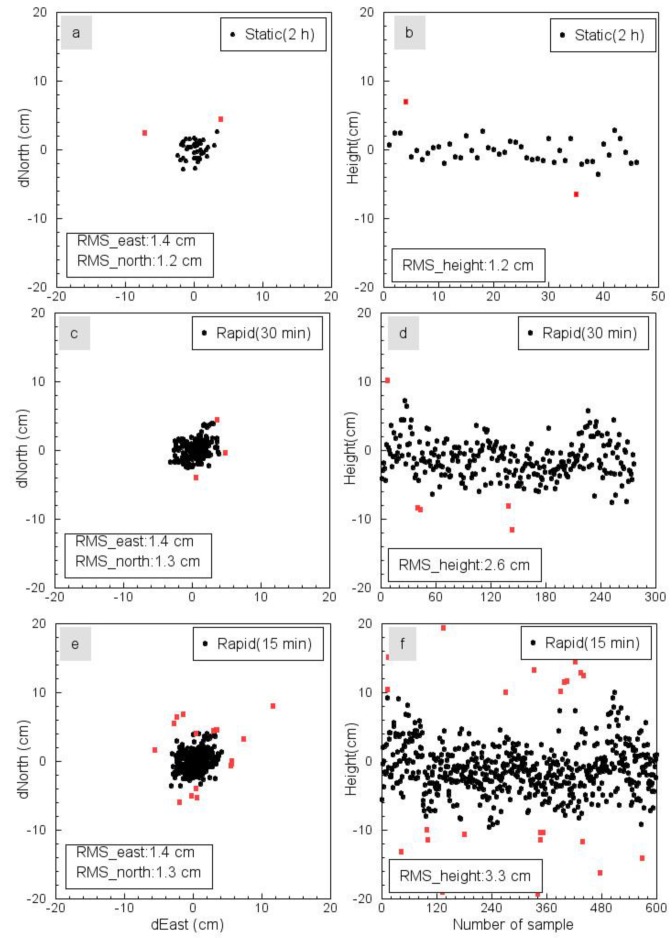
Plots illustrating the performance of the Online Positioning User Service for Static (OPUS-S) processing and Rapid-Static (OPUS-RS) processing in Freeport, Texas. (**a**) Horizontal trajectory of OPUS-S solutions for 46 2-h static GPS surveys at the benchmark (Figure 7); (**b**) heights of OPUS-S solutions for these 46 2-h static surveys; (**c**) and (**d**): horizontal and vertical solutions of OPUS-RS solutions for 276 30-min static GPS surveys at the benchmark; (**e**) and (**f**): horizontal and vertical solutions of OPUS-RS solutions for 618 15-min static GPS surveys at the benchmark. Red dots represent outliers that are not included in calculating the RMS of errors. The repeated GPS campaign surveys at the benchmark were conducted over six years, from 2013 to 2018.

Figure 8 illustrates the comparisons of OPUS-S (2 h), OPUS-RS (30 min), and OPUS-RS (15 min) solutions. The mean coordinates of each group have been removed from the original ENH coordinates. The left column depicts the errors (uncertainties) of OPUS solutions in the horizontal directions, and the right column depicts the errors in the vertical direction. The root mean square (RMS) of errors indicates the repeatability (precision) of OPUS solutions. Certain outliers have been removed before calculating each RMS. Outliers in each component are defined as measurements that have corresponding residues (after removing a mean) three times larger than the standard deviation of their group measurements. The statistics of OPUS solutions are summarized in Table 2. The RMS-accuracy of OPUS-S (2 h) is below 1.5 cm in all three directions. The RMS-accuracy of OPUS-RS solutions for 15 to 30-min observations was approximately 1.5 cm in the horizontal directions and 3 cm in the vertical direction. In the horizontal direction, there were 16 outliers for the 15-min OPUS-RS solutions (618), three outliers for the 30-min OPUS-RS solutions (276), and two outliers for the OPUS-S solutions (46). In the vertical direction, there were 26 outliers for the 15-min OPUS-RS solutions, five outliers for the 30-min OPUS-RS Solutions, and two outliers for the OPUS-S solutions. It is clear that the number of outliers can be significantly reduced by extending field observations from 15 min to 30 min. Furthermore, all eight “aborting” 15-min submissions were successfully solved by extending observations to 30 min. 

### 3.2. Accuracy of Georeferenced TLS Points 

**Figure 9:**
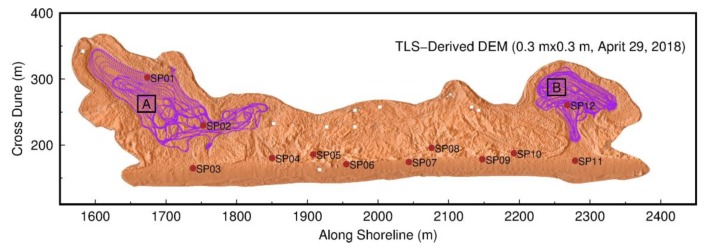
A hill-shaded digital elevation model (DEM) derived from the TLS datasets acquired on 29 April 2018, using the rapid surveying method. The location of the surveying area was marked in Figure 3b. The grid size was 0.3 m by 0.3 m. Red dots indicate 12 TLS scan positions. Purple dots represent kinematic GPS surveying points. Black rectangles indicate the areas that TLS and GPS points are compared in detail.

**Figure 10:**
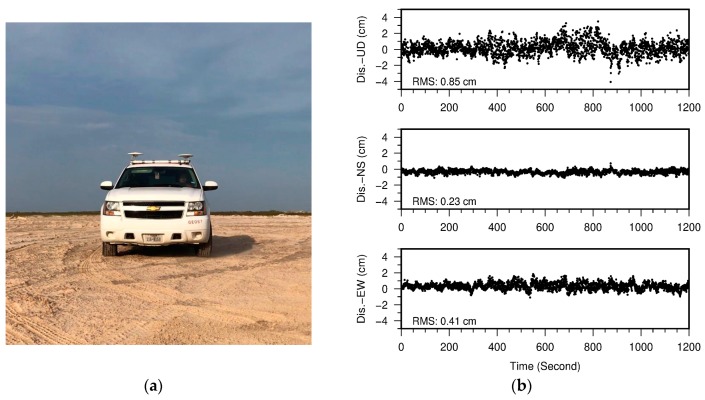
(**a**) Two GPS antennas mounted on the roof of a vehicle for kinematic GPS mapping; (**b**) three-component positional time series of the right antenna during a 20 min period when the vehicle remained stationary on the beach. The sampling rate was one sample per second (1 Hz).

In order to assess the elevation-accuracy of the TLS points georeferenced by the rapid surveying method, kinematic GPS surveys were performed in two washover-fans, as shown in Figure 9, where the ground surface is relatively flat and smooth. The kinematic GPS mapping was conducted by utilizing a reference station fixed at the USGS benchmark (Figure 7) and two Trimble NetR9 units mounted on the roof of a vehicle (Figure 10a). The kinematic GPS data was post-processed using RTKLIB (http://www.rtklib.com), which is an open source package for precise Global Navigation Satellite Systems (GNSS) positioning. The performance of RTKLIB has been investigated by previous studies [42,43,44]. In general, RTKLIB is able to retain 1 cm vertical accuracy and better accuracy in the horizontal directions for short baselines (e.g., <2 km). Figure 10b illustrates 20 min three-component displacement time series derived from the kinematic GPS measurements when the vehicle was stationary in the beach area. The sampling rate was one sample per second. The RMS-accuracy of the horizontal and vertical components was below one centimeter. The positional accuracy of kinematic GPS measurements when the vehicle is moving could be slightly worse.

**Figure 11:**
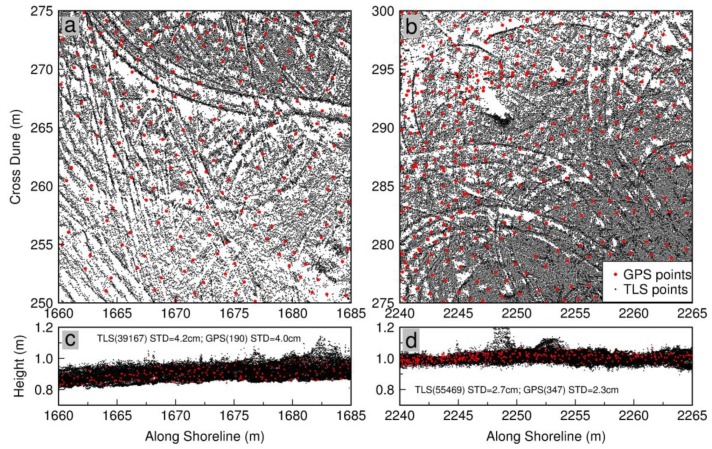
Comparisons of elevations (NAVD88) derived from georeferenced TLS points and kinematic GPS points. TLS datasets are collected with the rapid surveying method. (**a**) and (**b**): Locations of TLS and GPS surveying points within two 5 m by 5 m areas A and B, as marked in Figure 9; (**c**) and (**d**): elevation measurements from all TLS and GPS survey points in areas A and B, respectively.

As aforementioned, the vertical accuracy of the kinematic GPS measurements was below 1 cm. So, the GPS measurements can be regarded as true references to assess the elevation-accuracy of TLS measurements. Figure 11a,b depict the locations of TLS and kinematic GPS measurements within two 25 m by 25 m flat areas A and B, as marked in Figure 9. The surface of area B was smoother than the surface of area A. Figure 11c,d depict all elevations measured by kinematic GPS and TLS in areas A and B, respectively. Visually, the elevations from the two datasets agree very well in both areas. For area A, the mean elevation of 190 GPS measurements was 0.906 m, with a standard deviation (σ) of 4.0 cm; the mean elevation of 39,167 TLS measurements was 0.899 m, with a σ of 4.2 cm. For area B, the mean elevation of 347 GPS measurements was 1.003 m, with a σ of 2.3 cm; the mean elevation of 55,469 TLS measurements was 1.007 m, with a σ of 2.7 cm. The mean elevations of GPS and TLS measurements agree with each other at a few millimeters in both areas, which suggests that the georeferenced TLS measurements retain a similar elevation-accuracy as the kinematic GPS measurements for the specific datasets. The standard deviation on a flat surface indicates the scatter or precision (repeatability) of measurements. TLS measurements retain a slightly larger standard deviation in both areas, which suggests that the precision of TLS measurements may be slightly poorer than the precision of kinematic GPS measurements. It may be too optimistic to conclude that TLS measurements can achieve sub-centimeter elevation accuracy. However, the TLS and GPS datasets indeed demonstrate that carefully georeferenced TLS measurements have the ability to achieve substantially high elevation-accuracy in statistics. 

### 3.3. Accuracy of TLS-Derived DEMs

**Figure 12:**
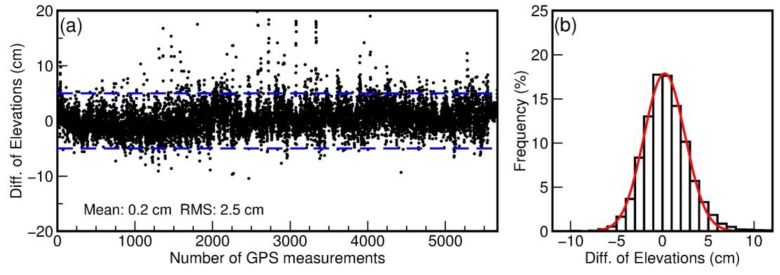
(**a**) Differences of elevations (NAVD88) between individual GPS points and their corresponding DEM grids derived from the georeferenced TLS points. (**b**) Histogram of elevation differences.

Bare earth DEMs are often regarded as final products of TLS topographic surveys. By default, the accuracy of TLS surveys is often referred to as the elevation-accuracy of DEMs rather than discrete laser points. The accuracy is often assessed by comparing the elevations of DEM grids with kinematic GPS measurements. Figure 12a depicts the differences (errors) between elevations measured by the kinematic GPS measurements illustrated in Figure 11 and their corresponding TLS-derived DEM grids. In total, 11,336 GPS and TLS pairs were compared. Since the surfaces of the washover areas were predominately flat and smooth, the specific gridding methods and grid size did not exert considerable differences on DEMs. The mean of the elevation differences (errors) of these 11,336 GPS-TLS pairs was 2 mm, with a standard deviation (1σ) of 2.5 cm. Figure 12b illustrates a histogram depicting distributions of the errors. The red curve superimposed on the histogram represents a normal distribution with a mean of 0.2 cm and a standard deviation of 2.5 cm. Table 3 lists the statistics of the error distributions. Approximately 73% of samples (errors) are within the band from –2.5 cm to 2.5 cm (1σ); 95% samples are within the band from –5 to 5 cm (2σ), and 99% samples are within the band from –7.5 to 7.5 cm (2σ). The distributions match well with the 68–95–99.7 empirical rule. More accurately, 68.27%, 95.45%, and 99.73% of values lie within one, two, and three standard deviations of the mean, respectively, for a normal distribution. The bell-shaped curve of the histogram and the error-distribution statistics suggest that the elevation errors of TLS points fit a normal distribution. 

**Figure 13:**
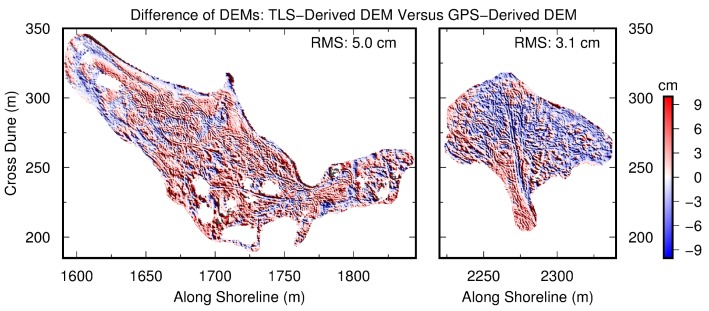
Map showing the difference between the TLS-derived digital elevation model (DEM) and GPS-derived DEM within two washover-fans marked in Figure 9. The grid size is 0.3 m by 0.3 m.

As mentioned earlier, the vertical RMS-accuracy (uncertainty) of OPUS-RS solutions for 15 to 25-min sessions is also at a level of 2 to 3 cm (Figure 8d,f). Thus, the vertical accuracy of the rapid TLS surveys is dominated by the vertical accuracy of GPS measurements on the scanner and the reflector. In other words, the two onboard inclination sensors may contribute only a small fraction of the total error budget of the TLS surveys. Of course, the effects of inclination sensors get larger with an increase in the distance to the scanner. Figure 13 depicts the differences of two DEMs (0.3 m by 0.3 m) derived from the kinematic GPS and TLS measurements, respectively. Identical procedures were used in generating the GPS-derived and TLS-derived DEMs. The RMS of the differences between two DEMs was 5 cm for the left washover-fan and 3 cm for the right washover-fan. The larger RMS of the left washover-fan was caused by its rougher beach surface. In summary, the elevation-accuracy of TLS-derived DEMs is at a level of approximately 3 cm.

### 3.4. Rapid Versus Conventional TLS Surveys

As aforementioned, the essence of registration and georeferencing is the seven-parameter coordinate transformation, which assumes that the crust block covered by the reference points is rigid. Nevertheless, certain coordinate distortions will be induced during each coordinate transformation because the surveying block is not strictly rigid. The distortion can be significant if the transformation utilizes a few reflectors while covering a large area. The conventional surveying method uses approximately five reflectors with GPS measurements (GPS-reflectors) to transform the PROC coordinates of the whole study area to a regional geodetic reference frame. As a consequence, the conventional surveying method often produces considerable coordinate distortions (errors), particularly in the areas where the distribution of GPS-reflectors is sparse. However, the rapid surveying method introduced in this article directly transforms point clouds from individual scans that cover a small area to a regional geodetic coordinate system. Thus, the rapid method avoids or minimizes coordinate distortions during coordinate transformations. 

**Figure 14:**
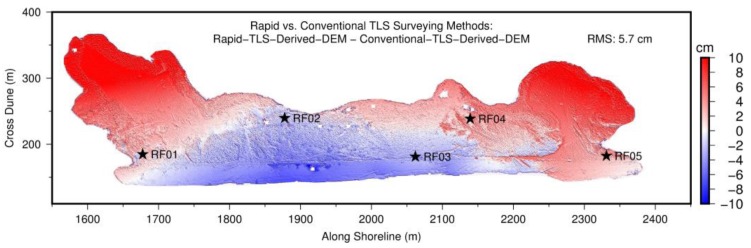
The difference between two DEMs derived from TLS datasets collected by the rapid surveying method and the conventional surveying method. Stars represent the locations of five reflectors with co-mounted GPS units (GPS-reflectors) that were used for georeferencing by the conventional surveying method.

Figure 14 depicts the differences between two DEMs (DDEM) derived from TLS datasets acquired with two different surveying methods: the rapid surveying method introduced in this study and the conventional surveying method. The conventional method used ten reflectors and five GPS-reflectors in the field. The locations of these reflectors are marked in Figure 4a. The datasets from 12 individual scans were registered to the XYZ-coordinate system aligned to the SOCS of the first scan position, then transformed to the ENH coordinate system using GPS measurements from five reflectors. The locations of these five GPS units mounted on reflectors are marked in Figure 14 as stars. Figure 14 indicates that these two DEMs agree with each other well (light red and light blue) in the beach and foredune areas, where most reflectors and GPSs were located. However, there were more substantial differences (deep red, >5 cm) at the northern edges of these two washover-fans, where few reflectors and no GPSs were located. As aforementioned, these significant differences were caused by the well-known coordinate distortion problem involved in the conventional surveying method. 

The spatial distribution and numbers of tie points (reflectors) are critically important for aligning two coordinate systems. According to our experiences from landslide projects, georeferenced TLS points, as well as DEMs, do not retain a consistent or homogeneous elevation accuracy in space [2,18,45]. Thus, it is a challenge to compare the accuracy of conventional and rapid surveying methods in a general way. However, the comparison presented in Figure 14 does suggest that the rapid surveying method can retain more consistent accuracy over space than the conventional surveying method. This is one of the advantages of the rapid surveying method.

### 3.5. TLS-Derived Topographic Changes

**Figure 15:**
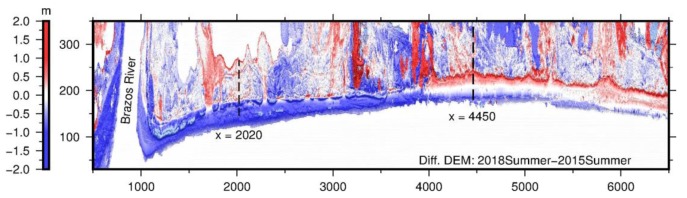
The difference between two DEMs (DDEM) derived from the TLS survey datasets in the summers of 2015 and 2018. The blue areas indicate sand loss (beach and dune erosion) and the red areas indicate sand gain (beach and dune growth).

**Figure 16:**
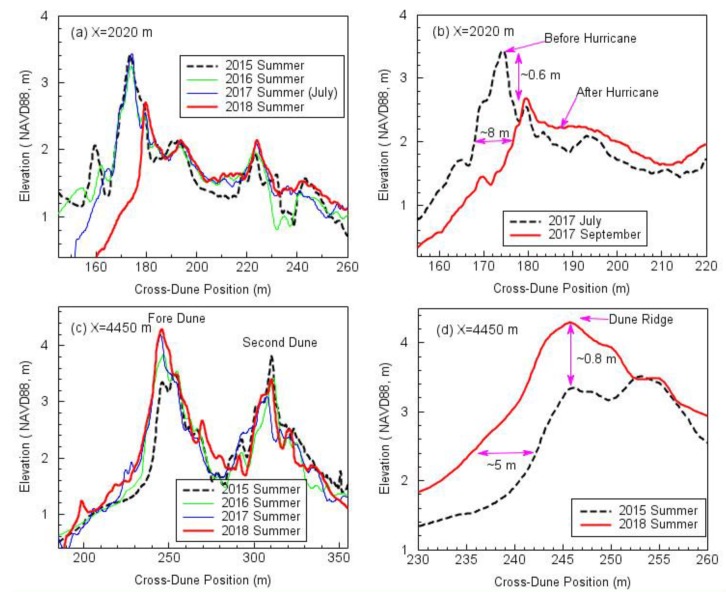
Cross-dune profiles extracted from DEMs (0.3 m by 0.3 m) obtained from rapid TLS surveys in four summers. (**a**) The cross-dune profiles at X = 2020 m; (**b**) the cross-dune profiles depicting significant dune erosions during the 2017 Hurricane Harvey; (**c**) the cross-dune profiles at X = 4450 m; (**d**) the cross-dune profiles depicting dune growth during a three-year period. The locations of the profiles are marked in Figure 15.

A map depicting the differences between two DEMs acquired at different times is often called DDEM. DDEM and topographic profiles are highly effective in depicting topographic changes (e.g., coastal erosion, landslide) over time and space [45,46,47]. The accuracy of elevation changes derived from a DDEM map could be better than the elevation-accuracy of original DEMs, since certain common errors that occur in generating DEMs have been removed by the differential process [45]. Figure 15 depicts the DDEM within the 7 km-long beach–dune area over three years from the summers of 2015 to 2018. The TLS datasets were collected by the rapid surveying method. The DDEM map clearly indicates that the beach and foredune areas adjacent to the Brazoria River lost massive sands (deep blue), while the beach and dune areas further away from the river gained sands (light red) during the three-year period. Figure 16 depicts cross-dune profiles at two locations, extracted from DEMs surveyed at different times: summers of 2015, 2016, 2017, and 2018. The locations of the profiles (X = 2020 m; X = 4450 m) are marked in Figure 15. Figure 16a,b indicate significant beach and dune erosion (deep blue), which was caused by a flood from the Brazos River during the 2017 Hurricane Harvey season (24 August–2 September). Figure 16c,d indicate that the foredune areas, approximately 3 km west from the river, did grow during this period. The front face of the foredune moved approximately 5 m toward the ocean, and the dune ridge grew approximately 0.8 m during the three-year period. 

It appears that the DDEM map and cross-dune profiles did a great job of quantitatively depicting the coastal topographic changes over space and time. However, TLS data users need to realize that the elevation-accuracy of a DEM is a function of the density and accuracy of survey points, gridding resolution, method of interpolation, and the complexity (or roughness) of terrain features. Data gaps and rough terrains can significantly degrade the accuracy of DEMs [48,49]. The grid size of DEMs is set as 0.3 m by 0.3 m (footprint) in this study. According to the Nyquist–Shannon sampling theorem [50,51], a sampled dataset with a grid spacing of “dx” cannot reconstruct ground features with wavelengths smaller than two times “dx”. Accordingly, researchers should be careful in explaining minor oceanward (growing) or landward (erosion) movements of dune ridges and dune toes. In order to precisely capture minor dune changes during a short period, a smaller grid size may be utilized. It is critical for non-expert TLS data users to understand the limits of DEMs. 

## 4. Discussion

LiDAR techniques have resulted in a new era of micro-topography analysis, using dense topographic measurements over a large geographic area. This study introduced a rapid TLS surveying method that takes advantage of onboard inclination sensors equipped with model laser scanners. According to this investigation, the overall accuracy of the georeferenced TLS points is dominated by the positional accuracy of the GPS antennas on the scanner and reflector; the onboard inclination sensors contribute a minor fraction of the total error budget. 

We proposed to use OPUS-S and OPUS-RS for GPS data post-processing. The accuracy of OPUS-RS solutions strongly depends on the length of the occupation period in the field. According to this study, OPUS-RS solutions (15 to 25 min observations) would retain a 1.5-cm horizontal accuracy and a 2 to 3-cm vertical accuracy in the Texas coastal region. For surveyors who want to further improve the positional accuracy of GPS and, in turn, the accuracy of TLS surveys, the advice is quite simple: slow down the scanning process and collect a longer period of GPS data at the scanning site. A 40-min GPS data session will be able to considerably improve the positional accuracy compared to a 20-min data session, particularly in the vertical direction [26,38]. OPUS-RS may not be able to solve a GPS position in coastal areas if OPUS does not have enough qualified CORS in this region during the surveying period. Users may consider using a commercial software package to process GPS data in the case that OPUS-RS does not work or works poorly in their study areas. However, particular attention should be paid to the details of reference frames and coordinate transformations.

## 5. Conclusions

TLS and GPS integrated technologies have provided practicing geologists with a new way to map the Earth’s surface at an unprecedented spatial resolution and accuracy over large areas and long-time spans, which is opening new ways of investigating coastal erosion problems. Many key scientific questions require high-resolution, high-accuracy, and repeated topographic datasets. These standards demand creative and well-designed data acquisition and processing procedures. This article introduced a rapid TLS surveying method for high-resolution (handprint) and high-accuracy (<5 cm) topographic mapping. The surveying method utilized one scanner, two GPS units, and only one reflector in the field. A team of two field crews is able to conduct over 20 scans within an 8-h fieldwork time. The proposed rapid surveying method will benefit both researchers and professional surveyors who use TLS for high-accuracy terrain mapping and geomorphological studies. 

This investigation indicated that OPUS-RS can achieve 1 to 2 cm horizontal accuracy (RMS) and 2 to 3 cm vertical accuracy for static observations of approximately 30 min in the Texas coastal region. The rapid TLS surveys can achieve an elevation-accuracy (RMS) of approximately 3 to 5 cm for georeferenced points and approximately 2 to 3 cm for TLS-derived DEMs. The elevation errors superimposed into the georeferenced TLS points roughly fit a normal distribution.

Recently, mini unmanned aerial vehicles (mini-UAVs) bearing LiDAR and photogrammetry techniques have been demonstrated as powerful tools for topographic mapping in coastal regions [52,53,54,55]. However, in order to obtain centimeter-accuracy measuring points, numerous ground control points (GCPs) need to be surveyed by few-centimeter-accuracy kinematic GPS. It is often a time-consuming process to survey GCPs in the field, particularly for repeated surveys aiming to track topographic changes in a large area over several years. The proposed rapid TLS surveys can provide millions of high-accuracy GCPs within a short time and at a low cost. Thus, rapid, low-cost, high-resolution, and high-accuracy topographic mapping can be achieved by integrating the rapid TLS surveying method and mini-UAV-borne LiDAR and photogrammetry survey techniques. The authors are working on the integration of ground-based and mini-UAV-borne LiDAR and photogrammetry techniques for coastal mapping. 

## Figures and Tables

**Table 1:** Scanner-to-reflector distances derived from GPS and TLS measurements.

Scanner–Reflector Pair *	GPS-Derived Distance (m)	TLS-Derived Distance (m)	Difference of Two Distances (cm)
SP01−Ref.01	213.455	213.458	0.3
SP02−Ref.02	125.198	125.203	0.5
SP03−Ref.03	158.162	158.166	0.4
SP04−Ref.04	211.740	211.747	0.7
SP05−Ref.05	230.506	230.510	0.4
SP06−Ref.06	277.745	277.745	0
SP07−Ref.07	287.613	287.612	0.1
SP08−Ref.08	202.968	202.954	1.4
SP09−Ref.09	183.535	183.534	0.1
SP10−Ref.10	139.149	139.146	0.3
SP11−Ref.11	153.003	153.005	0.2
SP12−Ref.12	130.221	130.233	1.2
			**Average:** 0.4

^*^ Scanner positions are marked in Figure 4.

**Table 2:** Statistics of OPUS solutions for campaign surveys at the benchmark.

Duration	2 h	30 min	15 min
Submitted files	46	276	618
Aborted (failed)	0	0	8
Outliers(horizontal)	2	3	16
Outliers(vertical)	2	5	26
RMS-East (cm)	1.2	1.3	1.4
RMS-North (cm)	1.2	1.2	1.2
RMS-Height (cm)	1.4	2.5	3.2

**Table 3:** The distribution of differences (errors) between elevations derived from kinematic GPS measurements and TLS-derived DEMs.

Error Interval (cm)	Percent (%) *	Total Percent (%)		
		[−1σ, +1σ]	[−2σ, +2σ]	[−3σ, +3σ]
<−7.5	0.09	-	-	-
−7.5 to −5	0.77	-	-	99.11
-5 to −2.5	8.91	-	95.34	
−2.5 to 0	35.50	72.81		
0 to 2.5	37.31			
2.5 to 5	13.62	-		
5 to 7.5	3.00		-	
> 7.5	0.80	-	-	-

* The number of total samples is 11,336.
